# Relationship Between Risk Factors and Brain Reserve in Late Middle Age: Implications for Cognitive Aging

**DOI:** 10.3389/fnagi.2019.00355

**Published:** 2020-01-09

**Authors:** Bryan J. Neth, Jonathan Graff-Radford, Michelle M. Mielke, Scott A. Przybelski, Timothy G. Lesnick, Christopher G. Schwarz, Robert I. Reid, Matthew L. Senjem, Val J. Lowe, Mary M. Machulda, Ronald C. Petersen, Clifford R. Jack Jr., David S. Knopman, Prashanthi Vemuri

**Affiliations:** ^1^Department of Neurology, Mayo Clinic, Rochester, MN, United States; ^2^Department of Health Sciences Research, Mayo Clinic, Rochester, MN, United States; ^3^Department of Radiology, Mayo Clinic, Rochester, MN, United States; ^4^Department of Information Technology, Mayo Clinic, Rochester, MN, United States; ^5^Department of Psychiatry and Psychology, Mayo Clinic, Rochester, MN, United States

**Keywords:** brain reserve, cognitive aging, multimodal imaging, resilience, dynamic

## Abstract

**Background:**

Brain reserve can be defined as the individual variation in the brain structural characteristics that later in life are likely to modulate cognitive performance. Late midlife represents a point in aging where some structural brain imaging changes have become manifest but the effects of cognitive aging are minimal, and thus may represent an ideal opportunity to determine the relationship between risk factors and brain imaging biomarkers of reserve.

**Objective:**

We aimed to assess neuroimaging measures from multiple modalities to broaden our understanding of brain reserve, and the late midlife risk factors that may make the brain vulnerable to age related cognitive disorders.

**Methods:**

We examined multimodal [structural and diffusion Magnetic Resonance Imaging (MRI), FDG PET] neuroimaging measures in 50–65 year olds to examine the associations between risk factors (Intellectual/Physical Activity: education-occupation composite, physical, and cognitive-based activity engagement; General Health Factors: presence of cardiovascular and metabolic conditions (CMC), body mass index, hemoglobin A1c, smoking status (ever/never), CAGE Alcohol Questionnaire (>2, yes/no), Beck Depression Inventory score), brain reserve measures [Dynamic: genu corpus callosum fractional anisotropy (FA), posterior cingulate cortex FDG uptake, superior parietal cortex thickness, AD signature cortical thickness; Static: intracranial volume], and cognition (global, memory, attention, language, visuospatial) from a population-based sample. We quantified dynamic proxies of brain reserve (cortical thickness, glucose metabolism, microstructural integrity) and investigated various protective/risk factors.

**Results:**

Education-occupation was associated with cognition and total intracranial volume (static measure of brain reserve), but was not associated with any of the dynamic neuroimaging biomarkers. In contrast, many general health factors were associated with the dynamic neuroimaging proxies of brain reserve, while most were not associated with cognition in this late middle aged group.

**Conclusion:**

Brain reserve, as exemplified by the four dynamic neuroimaging features studied here, is itself at least partly influenced by general health status in midlife, but may be largely independent of education and occupation.

## Introduction

Brain health is difficult to quantify – other than the absence of cognitive or neurological disease or pathology. The health of other organs is more easily measureable. For example, cardiac health can be described in terms of left ventricular ejection fraction, cardiac index, or burden of coronary artery disease (Mosterd and Hoes, 2007; Paulus et al., 2007; Jefferson et al., 2010). Renal health can be monitored by glomerular filtration rate or serum creatinine (Traynor et al., 2006). There are established thresholds or stages of disease severity for both congestive heart failure and chronic kidney disease (Coresh et al., 2007; Mosterd and Hoes, 2007). In contrast, although several fundamental components of brain health have been described, such as brain reserve and cognitive reserve or resilience, they have not been widely quantified and utilized.

**FIGURE 1:**
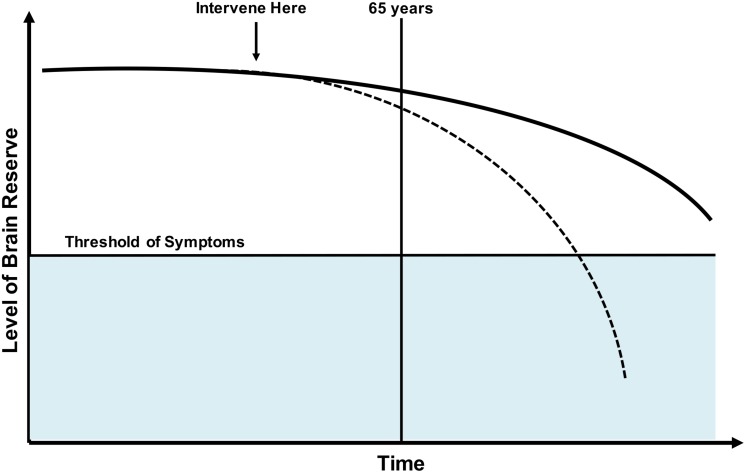
Model of brain reserve throughout the lifespan. Dotted line: Various factors may decrease brain reserve making likelihood of age-related cognitive disorders more likely. Solid line: Normal trajectory without onset of clinical symptoms. It would be ideal to study and intervene on factors that negatively influence brain reserve prior to onset of decline, with hopes of preventing or delaying onset of clinical disease.

The focus of the current study is on the concept of “brain reserve” or “neurobiological capital,” defined as individual brain variation that may lead to resistance or ability to cope with pathology (Stern et al., 2018). The traditionally used proxies of brain reserve include total intracranial volume, premorbid brain tissue volume, and head circumference (Stern et al., 2018), which are static or fixed in nature. Although each of these measures are a gross measure of the brain anatomic capital, these measures are not sufficient to define the overall brain reserve. Because midlife and late middle age represent a critical period where prominent aging-related brain changes begin (Debette et al., 2011; Ritchie et al., 2015), identifying alterations to brain reserve in this period will enhance the understanding of early changes in cognitive and brain aging. Furthermore, studying brain reserve in late middle age may provide insights into mechanisms of resilience that could contribute to a better accepted model of overall brain health (Arenaza-Urquijo and Vemuri, 2018; Stern et al., 2018, 2019). See Figure 1 for a model of brain reserve throughout life.

The main objective of this study was to broaden our understanding of brain reserve, protective/risk factors, and cognition in late middle age adult participants (age 50–65 years) without cognitive impairment. We focused on this age group because it is an age range during which both neuronal structure and functional alterations are observed but with few clinical symptoms (Giorgio et al., 2010; Jagust, 2013). We aimed to: (1) examine protective/risk factors of brain reserve measures and cognition; and (2) identify optimal neuroimaging measures related to global and domain-specific cognition that may best serve as dynamic neuroimaging biomarkers of brain reserve.

## Materials and Methods

### Selection of Participants

Study participants were from the Mayo Clinic Study of Aging (MCSA) (Roberts et al., 2008), an epidemiologic study of Mild Cognitive Impairment (MCI) and dementia among community-dwelling residents of Olmsted County, Minnesota. We included 537 late middle age participants (age 50–65 years) who had available Magnetic Resonance Imaging (MRI) data. A subset of 454 participants also had ^18^F-fluorodeoxyglucose positron emission tomography (FDG PET). All participants were cognitively unimpaired based upon a clinical adjudication at the clinical visit corresponding to the imaging visit. The MCSA was approved by the Mayo Clinic and Olmsted Medical Center Institutional Review Boards and all participants provided written informed consent.

### Measures of Brain Reserve

We utilized four dynamic or modifiable neuroimaging measures from three imaging modalities that are related to cognitive aging and dementia as proxies of overall brain reserve: genu corpus callosum fractional anisotropy (FA), posterior cingulate cortex FDG uptake, superior parietal cortex thickness, and AD signature cortical thickness. We term these proxies of brain reserve as dynamic because they are not constant or fixed across the adult lifespan like traditional brain reserve measures such as intracranial volume.

Diffusion tensor imaging (DTI) is a method utilized to quantify water diffusion throughout white matter tracts in the brain, with FA being one diffusion metric to assess white matter integrity (Le Bihan et al., 2001). Lower FA is related to less microstructural integrity of the white matter, and lower FA has been shown to be related to lower cognition in community-dwelling older adults (Vernooij et al., 2009) and throughout the Alzheimer’s spectrum (Bozzali et al., 2002; Zhang et al., 2007; Chua et al., 2008). Microstructural integrity of the genu corpus callosum as assessed by FA has been shown to be related of systemic vascular and cerebrovascular health (Vemuri et al., 2018), and is potentially an earlier surrogate of cerebrovascular health than white mater hyperintensities. There are intrinsic differences in myelination, axonal density, or even time to maturity of specific white matter tracks (Kochunov et al., 2012; Sexton et al., 2014) that uniquely differentiate the genu from other white matter tracks. Metabolism in the posterior cingulate cortex, one of the most metabolic brain regions, has been shown to preferentially decline early in preclinical Alzheimer’s disease and is lower in APOE E4 carriers (Cunnane et al., 2011; Protas et al., 2013; Leech and Sharp, 2014). Given the higher baseline glucose uptake of the posterior cingulate cortex relative to other brain regions and the disease-related metabolic decline of the posterior cingulate cortex, it may uniquely serve as proxy of brain health. Superior parietal cortex thickness has recently been related to systemic vascular health, such that greater thickness was associated with a higher number of vascular conditions. Greater thickness has been posited to be a compensatory response to early pathology (Vemuri et al., 2018). Reports have described higher parietal volume in amyloid positive participants (Johnson et al., 2014), and greater compensatory superior parietal cortical thickness in those with lower CSF amyloid prior to atrophy that coincides with increased CSF p-tau (Fortea et al., 2014). With the significant impact of systemic vascular health on the brain and potential influence of amyloid and tau on the superior parietal lobule, we believe superior parietal cortical thickness uniquely contributes to a more comprehensive view of brain health. We chose to include Alzheimer’s disease signature cortical thickness as a measure of brain reserve because it has been validated as a measure of neurodegeneration and is likely a better measure than other traditionally used proxies of age and disease related neurodegeneration, like hippocampal volume, as it is not confounded by head size (Jack et al., 2015).

#### Structural and Diffusion MRI

All MRI images were acquired on 3T GE MRI (GE Medical Systems, Milwaukee, WI, United States) using a Sagittal 3D magnetization prepared rapid acquisition gradient recalled echo (MP-RAGE) sequence. Repetition time (TR) was ≈2300 ms, echo time (TE) ≈3 ms, and inversion time (TI) = 900 ms. Voxel dimensions were ≈1.20 × 1.015 × 1.015 mm.

Cortical thickness measurements were computed using Freesurfer v5.3 and total intracranial volume was computed using a previously published method (Schwarz et al., 2016) on standard structural magnetization-prepared rapid acquisition gradient echo (MPRAGE) scans. We considered the dynamic measures of superior parietal cortex thickness and composite measure of cortical thickness from AD vulnerable regions (average of thickness in entorhinal cortex, inferior temporal, middle temporal, fusiform) (Jack et al., 2015). As a comparison to these measures, we have also performed analyses with the static measure of total intracranial volume.

The details of DTI acquisition and processing are discussed in our recent publication (Vemuri et al., 2018). We considered genu of the corpus callosum microstructural integrity as quantified by FA from DTI.

#### FDG PET

The acquisition, processing, and summary measure details for FDG PET scans acquired on the MCSA study participants are previously described (Jack et al., 2015). Computed tomography scan was obtained for attenuation correction and FDG PET images were obtained 30–40 min after tracer injection. We considered posterior cingulate cortex glucose metabolism from FDG PET.

### Selection of Protective and Risk Factors

We examined a broad range of protective and risk factors including traditionally viewed proxies for resilience (e.g., education, intellectual and physical activities) (Stern et al., 2018), and overall proxies of health (e.g., chronic or comorbid conditions).

#### Intellectual and Physical Activities

We utilized an education-occupation composite measure that incorporates years of education and job level score that is based on the participant’s primary occupation (Vemuri et al., 2015). We assessed physical and cognitive-based activity using a questionnaire that quantified the average activity in each domain during the last 12 months (Vemuri et al., 2012). In our sample of 50–65 years old participants, these represent self-reported measures of physical and cognitive-based activities at late middle age. A complete list of activities queried on the questionnaires are previously published (Vemuri et al., 2012).

#### General Health Measures

Given the relationships found between overall health and cognitive aging and/or age-related disease (Whitmer et al., 2005, 2008; Yaffe et al., 2006; Crooks et al., 2008; Craft, 2009; Byers and Yaffe, 2011), we included measures that are not routinely studied in the context of cognitive resilience. The presence of cardiovascular and metabolic conditions (CMC) is a measure composed of health system data, ICD-9 and ICD-10 codes of seven common conditions related to systemic health: hypertension, hyperlipidemia, cardiac arrhythmias, coronary artery disease, congestive heart failure, diabetes mellitus, and stroke (Vemuri et al., 2017, 2018). The CMC composite score is an additive measure of the absence or presence of each condition, with a range score of 0–7 (Vemuri et al., 2017, 2018). With increasing use of electronic medical records for research data, this metric may be derived from already collected data and serve as an overall metric of systemic cardiovascular/metabolic disease burden. In addition to CMC, we also studied body mass index [BMI, mass (kg)/height (m^2^)] (Calle et al., 1999), hemoglobin A1c (average blood glucose of around the last 90–120 days) (Rohlfing et al., 2002), ever-smoking (dichotomous), score on the CAGE Alcohol Questionnaire >2 (dichotomous) (Ewing, 1984), and continuous score on the Beck Depression Inventory (Beck et al., 1996).

### Measures of Cognition

As previously described, cognitive tests were administered by a psychometrist and included nine tests covering four domains: memory [WMS-R Logical Memory-II (delayed), WMS-R Visual Reproduction-II (delayed), AVLT (delayed)], attention (TMT: Part B, WAIS-R Digit Symbol), language (BNT, category fluency), and visuospatial (WAIS-R Picture Completion, WAIS-R Block Design) (Roberts et al., 2008). Individual test scores from each domain were converted into *z*-scores, which were then averaged to make domain-specific *z*-scores. Global cognition was estimated from the average of the four domain-specific *z*-scores and then itself converted into a *z*-score for analyses.

### Statistical Analyses

We performed multivariable linear regression to examine the relationship between: (1) protective/risk factors and brain reserve measures, (2) brain reserve measures and cognition, (3) protective/risk factors and cognition. Next, to relate both brain reserve measures and protective/risk factors independently to cognition, each brain reserve measure and protective/risk factor was used as a predictor in regression models. All analyses were adjusted for age, sex, and the presence of an APOE E4 allele. We also performed *t*-test and chi-square analyses to assess for mean differences between sexes in cross-sectional protective/risk factors, brain reserve measures, and cognition. SAS University Edition was utilized for analyses. A *p* < 0.05 was considered statistically significant.

## Results

Participant characteristics are shown in Table 1. Our sample included 537 participants with a mean age of 58.7 years. There were nearly identical number of females and males (269 and 268). Of the 537 participants, 29.1% had an APOE4 allele. Mean education was 15.2 years with a range between 9–20 years.

**TABLE 1:** Descriptive statistics for total sample.

**Variable**	**All (*n* = 537)**
Age (years)	58.7 (4.3)
Education (years)	15.2 (2.2)
Educ-occ composite	13.1 (2.2)
Global cognition (*Z*-score)	0.75 (0.77)
Memory (*Z*-score)	0.62 (0.88)
Attention (*Z*-score)	0.66 (0.74)
Language (*Z*-score)	0.50 (0.87)
Visuospatial (*Z*-score)	0.61 (0.84)
Genu corpus callosum (FA)	0.62 (0.04)
Posterior cingulate (FDG)	1.96 (0.16)
AD ROI (Thick)	2.98 (0.12)
Superior parietal (Thick)	2.04 (0.13)
Intracranial volume	1488.5 (161.7)
CMC	1.0 (1.1)
BMI	29.3 (5.6)
HbA1c	5.6 (0.7)
BDI score	4.1 (4.8)
Physical activity	6.7 (4.6)
Cognitive activity	21.1 (8.6)
Sex: F/M (%)	269(50)/268(50)
Smoke: No/Yes (%)	324(60)/213(40)
CAGE > 2: No/Yes (%)	500(93)/37(7)
APOE4: −/+ (%)	370(71)/152(29)

*Mean (Standard Deviation) for continuous variables. Count (%) for dichotomous variables.*

Table 2 shows descriptive statistics between females and males. There were no differences in age, education years, APOE4 status between males and females. Females had higher global cognition, memory, attention, language performance; males had higher visuospatial skills. Females had higher posterior cingulate FDG and superior parietal thickness, despite lower intracranial volume and genu FA. Females had lower presence of CMC and higher self-reported cognitive activity engagement. There were no sex differences in body mass index, HbA1c, smoking status, Beck Depression Inventory score, or CAGE score.

**TABLE 2:** Descriptive statistics by sex.

**Variable**	**Female (*n* = 269)**	**Male (*n* = 268)**	***p*-value**
Age (years)	58.7 (4.3)	58.7 (4.2)	ns
Education (years)	15.1 (2.2)	15.3 (2.2)	ns
Educ-occ composite	12.8 (2.3)	13.3 (2.1)	0.0195
Global cognition (*Z*-score)	0.83 (076)	0.68 (0.77)	0.0297
Memory (*Z*-score)	0.78 (0.84)	0.45 (0.88)	<0.0001
Attention (*Z*-score)	0.79 (0.75)	0.53 (0.70)	<0.0001
Language (*Z*-score)	0.60 (0.89)	0.39 (0.84)	0.0049
Visuospatial (*Z*-score)	0.45 (0.80)	0.78 (0.85)	<0.0001
Genu corpus callosum (FA)	0.62 (0.04)	0.63 (0.04)	0.0269
Posterior cingulate (FDG)	2.00 (0.16)	1.92 (0.16)	<0.0001
AD ROI (Thick)	2.98 (0.12)	2.98 (0.12)	ns
Superior parietal (Thick)	2.06 (0.12)	2.03 (0.13)	0.0013
Intracranial volume	1381.3 (112.9)	1596.1 (128.4)	<0.0001
CMC	1.0 (1.1)	1.3 (1.2)	0.0003
BMI	29.3 (6.6)	29.3 (4.5)	ns
HbA1c	5.5 (0.6)	5.6 (0.8)	ns
BDI score	4.1 (4.7)	4.7 (4.9)	ns
Physical activity	6.6 (4.3)	6.8 (4.8)	ns
Cognitive activity	23.3 (8.6)	18.9 (8.1)	<0.0001
Smoke: No/Yes (%)	170(63)/99(37)	154(57)/114(43)	ns
CAGE > 2: No/Yes (%)	255(94)/14(6)	245(91)/23(9)	ns
APOE4: −/+ (%)	187(72)/74(28)	183(70)/78(30)	ns

*Mean (Standard Deviation) for continuous variables. Count (%) for dichotomous variables. *p*-values for *t*-test and chi-square of differences in group means.*

### Relationship Between Protective/Risk Factors and Brain Reserve Measures

**FIGURE 2:**
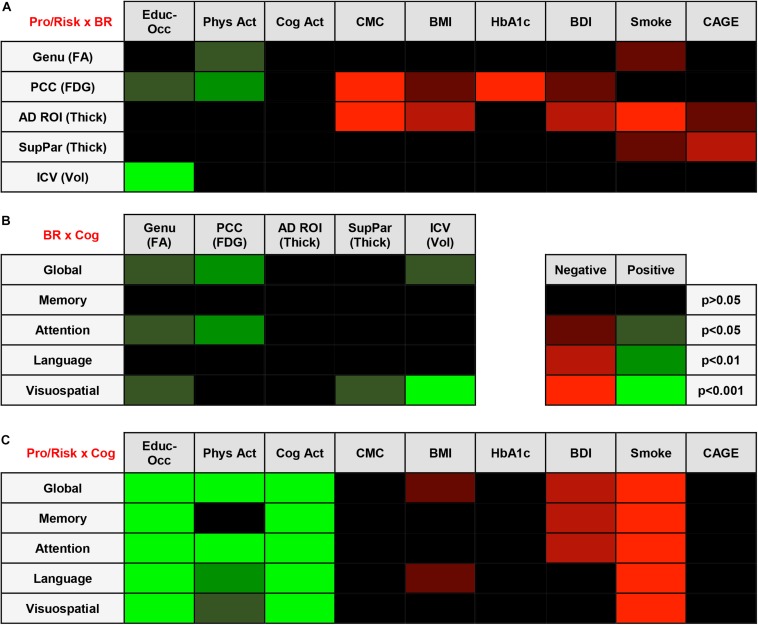
**(A)** Regression Heatmap for Protective/Risk Factors × Brain Reserve Measures. Adjusted analyses shown in figure. Shades of green indicate positive relationships between Protective/Risk Factors and Brain Reserve Measures. Shades of red indicate negative relationships between Protective/Risk Factors and Brain Reserve Measures. Complete regression output with Beta and SE can be found in Table 3A. **(B)** Regression Heatmap for Brain Reserve Measures × Cognitive Measures. Adjusted analyses shown in figure. Shades of green indicate positive relationships between Brain Reserve Measures and Cognitive Measures. There were no negative relationships between Brain Reserve and Cognitive Measures. Complete regression output with Beta and SE can be found in Table 3B. **(C)** Regression Heatmap for Protective/Risk Factors × Cognitive Measures. Adjusted analyses shown in figure. Shades of green indicate positive relationships between Protective/Risk Factors and Cognitive Measures. Shades of red indicate negative relationships between Protective/Risk Factors and Cognitive Measures. Complete regression output with Beta and SE can be found in Table 3C.

A regression heatmap from analyses adjusted for age, sex, and APOE E4 can be found in Figure 2A, and complete regression output can be found in Table 3A. A higher education-occupation composite score was associated with higher posterior cingulate cortex FDG uptake and greater intracranial volume. More physical activity was associated with higher genu FA and posterior cingulate cortex FDG uptake. We found no associations between cognitive-based activity and any of the brain reserve measures.

**TABLE 3:** Full regression output including Beta, SE, and *p*-values for tested relationships, emboldened results are significant.

**(A)**																											
																											
**Unadjusted**	**Educ-Occ**			**Phys Act**			**Cog Act**			**CMC**			**BMI**			**HbA1c**			**BDI**			**Smoke**			**CAGE**		
									
**Risk × BR**	**B**	**SE**	**p**	**B**	**SE**	**p**	**B**	**SE**	**p**	**B**	**SE**	**p**	**B**	**SE**	**p**	**B**	**SE**	**p**	**B**	**SE**	**p**	**B**	**SE**	**p**	**B**	**SE**	**p**
																											
**GCC (FA)**	0.00108	0.0007	0.1362	**0.0008**	**0**.**0003**	**0.01780**	–0.00025	0.0002	0.1744	−**0.00347**	**0**.**0014**	**0.01060**	–0.00023	0.0003	0.4138	–0.00153	0.0024	0.5255	–0.00026	0.0003	0.4312	−**0.00683**	**0.0032**	**0.03520**	–0.00512	0.0063	0.4148
**PCC (FDG)**	0.00481	0.0035	0.168	**0.0046**	**0**.**0017**	**0.00620**	**0.00240**	**0**.**0009**	**0.00720**	−**0.03078**	**0**.**0064**	**0.00001**	−**0.00285**	**0**.**0014**	**0.03550**	−**0.04327**	**0**.**0112**	**0.00010**	−**0.00415**	**0**.**0016**	**0.01150**	−**0.03349**	**0.0157**	**0.03330**	–0.00012	0.0304	0.9969
**AD ROI (Thick)**	0.00321	0.0024	0.19	0.0023	0.0012	0.0502	–0.00021	0.0006	0.7337	−**0.02029**	**0**.**0045**	**0.00001**	−**0.00259**	**0**.**0010**	**0.00680**	–0.00838	0.0081	0.3022	−**0.00319**	**0**.**0011**	**0.00430**	−**0.03970**	**0.0108**	**0.00030**	−**0.05290**	**0.0211**	**0.01230**
**SupPar (Thick)**	0.00255	0.0025	0.314	0.0001	0.0012	0.9613	0.00105	0.0006	0.1035	–0.00384	0.0047	0.4184	0.00173	0.0010	0.0809	–0.00654	0.0084	0.4353	–0.00069	0.0012	0.5499	−**0.02731**	**0.0112**	**0.01550**	−**0.07410**	**0.0216**	**0.00070**
**ICV (Vol)**	**14.53234**	**3**.**1316**	**0.00001**	1.7694	1.5417	0.2516	−**2.21202**	**0**.**8103**	**0.00650**	10.88245	5.9642	0.0686	–1.92489	1.2462	0.123	–6.35487	10.6098	0.5495	0.27990	1.4600	0.848	–1.28664	14.2745	0.9282	33.04846	27.5332	0.2305
																											

**Adjusted**	**Educ-Occ**			**Phys Act**			**Cog Act**			**CMC**			**BMI**			**HbA1c**			**BDI**			**Smoke**			**CAGE**		
									
**Risk × BR**	**B**	**SE**	**p**	**B**	**SE**	**p**	**B**	**SE**	**p**	**B**	**SE**	**p**	**B**	**SE**	**p**	**B**	**SE**	**p**	**B**	**SE**	**p**	**B**	**SE**	**p**	**B**	**SE**	**p**

																											
**GCC (FA)**	0.00091	0.0007	0.2006	**0.0007**	**0**.**0003**	**0.04770**	–0.00012	0.0002	0.5285	–0.00242	0.0014	0.0806	–0.00019	0.0003	0.4906	–0.00112	0.0024	0.6352	–0.00035	0.0003	0.2799	−**0.00621**	**0.0032**	**0.04990**	–0.00543	0.0061	0.375
**PCC (FDG)**	0.00756	0.0033	0.02380	**0.0045**	**0**.**0016**	**0.00580**	0.00137	0.0009	0.1243	−**0.02128**	**0**.**0064**	**0.00100**	−**0.00280**	**0**.**0013**	**0.03050**	−**0.03754**	**0**.**0108**	**0.00060**	−**0.00398**	**0**.**0016**	**0.01100**	–0.02530	0.0150	0.0929	0.00935	0.0290	0.7475
**AD ROI (Thick)**	0.00323	0.0024	0.1867	0.0020	0.0012	0.0842	–0.00018	0.0006	0.7814	−**0.01840**	**0**.**0047**	**0.00010**	−**0.00252**	**0**.**0009**	**0.00820**	–0.00701	0.0081	0.3871	−**0.00327**	**0**.**0011**	**0.00320**	−**0.03778**	**0.0108**	**0.00050**	−**0.05173**	**0.0210**	**0.01400**
**SupPar (Thick)**	0.00336	0.0025	0.1797	–0.0001	0.0012	0.9073	0.00064	0.0007	0.3265	0.00214	0.0049	0.6606	0.00179	0.0010	0.0665	–0.00394	0.0083	0.6348	–0.00056	0.0011	0.6276	−**0.02319**	**0.0111**	**0.03770**	−**0.06849**	**0.0213**	**0.00140**
**ICV (Vol)**	**9.67513**	**2**.**3652**	**0.00001**	1.2796	1.1553	0.2685	1.08832	0.6315	0.0854	–2.91924	4.6813	0.5332	–1.81222	0.9321	0.0524	–14.39758	8.0080	0.0728	–1.13769	1.0918	0.2979	–13.81308	10.7057	0.1975	5.07107	20.6777	0.8064

**(B)**																											

																											
**Unadjusted**	**GCC**			**PCC**			**AD ROI**			**SupPar**			**ICV**														
																	
**BR × Cog**	**B**	**SE**	**p**	**B**	**SE**	**p**	**B**	**SE**	**p**	**B**	**SE**	**p**	**B**	**SE**	**p**												

																											
**Global**	**2.80062**	**0**.**9010**	**0.00200**	**0.9593**	**0**.**2262**	**0.00001**	0.45760	0.2720	0.0931	0.44047	0.2621	0.0934	0.00003	0.0002	0.8902												
**Memory**	1.08056	1.0296	0.2944	**0.6980**	**0**.**2449**	**0.00460**	0.23238	0.3054	0.4471	–0.11163	0.2964	0.7066	−**0.00074**	**0**.**0002**	**0.00160**												
**Attention**	**2.24928**	**0**.**8604**	**0.00920**	**1.0748**	**0**.**2169**	**0.00001**	0.40709	0.2576	0.1146	**0.63481**	**0**.**2478**	**0.01070**	–0.00026	0.0002	0.1876												
**Language**	**2.02338**	**1**.**0206**	**0.04790**	**0.7009**	**0**.**2514**	**0.00550**	0.14444	0.3050	0.636	0.29686	0.2955	0.3156	–0.00009	0.0002	0.7143												
**Visuospatial**	**3.60372**	**0**.**9785**	**0.00030**	0.2285	0.2447	0.351	**0.68096**	**0**.**2952**	**0.02150**	**0.63359**	**0**.**2838**	**0.02600**	**0.00129**	**0**.**0002**	**0.00001**												
																											

**Adjusted**	**GCC**			**PCC**			**AD ROI**			**SupPar**			**ICV**														
																	
**BR × Cog**	**B**	**SE**	**p**	**B**	**SE**	**p**	**B**	**SE**	**p**	**B**	**SE**	**p**	**B**	**SE**	**p**												

																											
**Global**	**2.11878**	**0**.**9084**	**0.02010**	**0.6834**	**0**.**2342**	**0.00370**	0.37298	0.2730	0.1725	0.21459	0.2605	0.4104	**0.00054**	**0**.**0003**	**0.04660**												
**Memory**	1.05430	1.0379	0.3102	0.4135	0.2547	0.25466	0.13757	0.3017	0.6486	–0.39130	0.2949	0.1851	–0.00013	0.0003	0.6776												
**Attention**	**1.93948**	**0**.**8550**	**0.02370**	**0.7098**	**0**.**2224**	**0.00150**	0.22823	0.2487	0.3593	0.35902	0.2425	0.1393	0.00050	0.0003	0.0511												
**Language**	1.86428	1.0403	0.0737	0.4275	0.2617	0.1031	0.05065	0.3040	0.8677	0.09951	0.2976	0.7382	**0.00061**	**0**.**0003**	**0.0496**												
**Visuospatial**	**2.36245**	**0**.**9787**	**0.01610**	0.2611	0.2494	0.2955	0.52470	0.2874	0.0685	**0.68476**	**0**.**2784**	**0.01420**	**0.00105**	**0**.**0003**	**0.00030**												

**(C)**																											

																											
**Unadjusted**	**Educ-Occ**			**Phys Act**			**Cog Act**			**CMC**			**BMI**			**HbA1c**			**BDI**			**Smoke**			**CAGE**		
									
**Risk × Cog**	**B**	**SE**	**p**	**B**	**SE**	**p**	**B**	**SE**	**p**	**B**	**SE**	**p**	**B**	**SE**	**p**	**B**	**SE**	**p**	**B**	**SE**	**p**	**B**	**SE**	**p**	**B**	**SE**	**p**

																											
**Global**	**0.13989**	**0**.**0143**	**0.00001**	**0.0274**	**0**.**0074**	**0.00020**	**0.02990**	**0**.**0037**	**0.00001**	−**0.06770**	**0**.**0287**	**0.01880**	−**0.01272**	**0**.**0060**	**0.03440**	0.01142	0.0505	0.8211	−**0.02037**	**0**.**0069**	**0.00350**	−**0.39435**	**0.0668**	**0.00001**	–0.06607	0.1332	0.62
**Memory**	**0.10201**	**0**.**0168**	**0.00001**	0.0155	0.0084	0.0655	**0.02460**	**0**.**0043**	**0.00001**	–0.03664	0.0326	0.2615	–0.01009	0.0067	0.1349	0.00809	0.0568	0.8868	−**0.02636**	**0**.**0079**	**0.00080**	−**0.32235**	**0.0763**	**0.00001**	–0.29116	0.1487	0.0508
**Attention**	**0.09022**	**0**.**0142**	**0.00001**	**0.0233**	**0**.**0070**	**0.00100**	**0.02630**	**0**.**0036**	**0.00010**	−**0.09937**	**0**.**0271**	**0.00030**	–0.01030	0.0057	0.0706	–0.01955	0.0485	0.6868	−**0.02044**	**0**.**0066**	**0.00200**	−**0.33217**	**0.0638**	**0.00001**	–0.06403	0.1271	0.6147
**Language**	**0.14679**	**0**.**0162**	**0.00001**	**0.0260**	**0**.**0083**	**0.00180**	**0.03322**	**0**.**0042**	**0.00001**	–0.04264	0.0324	0.189	−**0.01393**	**0**.**0067**	**0.03850**	0.00327	0.0574	0.9546	–0.01463	0.0079	0.0638	−**0.35482**	**0.0759**	**0.00001**	–0.00463	0.1482	0.14823331
**Visuospatial**	**0.11419**	**0**.**0161**	**0.00001**	**0.0189**	**0**.**0081**	**0.01940**	**0.01202**	**0**.**0043**	**0.00500**	–0.01840	0.0314	0.5581	–0.00248	0.0066	0.7058	0.05340	0.0549	0.3309	–0.00166	0.0076	0.8283	−**0.26879**	**0.0740**	**0.00030**	0.06110	0.1455	0.6747
																											

**Adjusted**	**Educ-Occ**			**Phys Act**			**Cog Act**			**CMC**			**BMI**			**HbA1c**			**BDI**			**Smoke**			**CAGE**		
									
**Risk × Cog**	**B**	**SE**	**p**	**B**	**SE**	**p**	**B**	**SE**	**p**	**B**	**SE**	**p**	**B**	**SE**	**p**	**B**	**SE**	**p**	**B**	**SE**	**p**	**B**	**SE**	**p**	**B**	**SE**	**p**

																											
**Global**	**0.14417**	**0**.**0138**	**0.00001**	**0.0251**	**0**.**0072**	**0.00050**	**0.03064**	**0**.**0038**	**0.00001**	–0.02388	0.0295	0.4185	−**0.01250**	**0**.**0058**	**0.03270**	0.03165	0.0494	0.5221	−**0.02046**	**0**.**0068**	**0.00270**	−**0.36405**	**0.0655**	**0.00001**	–0.02553	0.1302	0.8446
**Memory**	**0.11046**	**0**.**0164**	**0.00001**	0.0144	0.0082	0.0808	**0.02132**	**0**.**0044**	**0.00001**	0.00635	0.0335	0.8497	–0.00985	0.0066	0.1361	0.02933	0.0558	0.5996	−**0.02483**	**0**.**0077**	**0.00140**	−**0.29354**	**0.0752**	**0.00010**	–0.24288	0.1461	0.0969
**Attention**	**0.09696**	**0**.**0136**	**0.00001**	**0.0237**	**0**.**0068**	**0.00050**	**0.02472**	**0**.**0035**	**0.00001**	–0.05286	0.0275	0.0551	–0.01014	0.0054	0.0621	0.00615	0.0464	0.8948	−**0.02001**	**0**.**0063**	**0.00160**	−**0.29355**	**0.0615**	**0.00001**	–0.06364	0.1248	0.6102
**Language**	**0.15326**	**0**.**0160**	**0.00001**	**0.0248**	**0**.**0082**	**0.00270**	**0.03292**	**0**.**0043**	**0.00001**	–0.00844	0.0337	0.8024	−**0.01367**	**0**.**0066**	**0.04000**	0.02159	0.0569	0.7046	–0.01386	0.0078	0.0765	−**0.33347**	**0.0755**	**0.00001**	0.02981	0.1469	0.8393
**Visuospatial**	**0.10693**	**0**.**0156**	**0.00001**	**0.0160**	**0**.**0078**	**0.04080**	**0.01919**	**0**.**0042**	**0.00001**	–0.00635	0.0317	0.8412	–0.00167	0.0063	0.7926	0.05236	0.0532	0.3254	–0.00468	0.0074	0.5271	−**0.26906**	**0.0716**	**0.00020**	0.03224	0.1407	0.8189

*Results from unadjusted analyses and analyses adjusted for age, sex, and APOE4-status are provided. **(A)** Protective/Risk Factors × Brain Reserve Measures, **(B)** Brain Reserve Measures × Cognitive Measures, **(C)** Protective/Risk Factors × Cognitive Measures. Bold values denote statistical significance at a threshold of *p* < 0.05.*

A higher number of CMC and higher Beck Depression Inventory scores were associated with lower posterior cingulate cortex FDG uptake and AD signature region thickness. Being an ever-smoker (relative to never-smoker) was associated with lower genu FA, AD signature region thickness, and superior parietal cortex thickness. CAGE Alcohol Questionnaire score >2 was associated with lower AD signature region thickness and superior parietal cortex thickness. Higher body mass index was related to lower posterior cingulate cortex FDG uptake and superior parietal cortex thickness, and higher HbA1c was negatively related to lower posterior cingulate cortex FDG uptake.

### Relationship Between Brain Reserve Measures and Cognition

We found that higher brain reserve measures were associated with better global and domain-specific cognition. A regression heatmap from adjusted analyses can be found in Figure 2B, and complete regression output can be found in Table 3B. Higher intracranial volume was associated with better global cognition and visuospatial ability; higher genu FA with better global cognition, attention, and visuospatial ability; higher posterior cingulate cortex FDG uptake with better global cognition and attention; and higher superior parietal thickness with better visuospatial ability. There were no significant associations between AD signature region thickness and cognition in multivariable models.

### Relationship Between Protective/Risk Factors and Cognition

Education-occupation composite score and cognitive-based activity engagement in the last 12 months were associated with better global and domain-specific cognition across all domains. Physical activity engagement in the last 12 months was associated with better global cognition and better cognition in attention, language, and visuospatial ability domain, but not memory. A regression heatmap from adjusted analyses can be found in Figure 2C, and complete regression output can be found in Table 3C.

Being an ever-smoker (relative to non-smoker) was associated with worse cognition across all domains. A greater number of depressive symptoms was associated with worse performance in global cognition and on tests of memory and attention. Higher body mass index was associated with worse global cognition and language. We found no relationship between HbA1c or CAGE Alcohol Questionnaire score >2 and global or domain-specific cognition.

## Discussion

We examined the relationships between protective/risk factors and imaging proxies of brain reserve in a late midlife cohort. Our major finding was that several general health factors were associated with worsening of the four dynamic neuroimaging biomarkers, in a manner that was not complicated by concomitant associations of declines in cognition and at least some of the general health factors. Depression and smoking showed associations with the dynamic neuroimaging proxies of reserve but also cognition, precluding any claims about their indirect relationships to brain reserve. Education-occupation was not associated with any of the dynamic brain imaging measures, but was associated with the static brain reserve proxy of total intracranial volume.

Brain reserve, as exemplified by the four dynamic imaging features studied here, is itself at least partly under the influence of general health status in midlife, but remarkably is largely independent of education and occupation. Health issues such as CMC, BMI, glycemic control and alcohol use that arise in midlife may have indirect effects on risks for later life cognition by influencing brain structure and function beginning in midlife or even earlier. Thus, white matter integrity in the genu corpus callosum, posterior cingulate cortex FDG, and cortical thickness are influenced by midlife health factors that in later life moderate the effects of age-related neurodegenerative and cerebrovascular diseases.

Several features distinguish this study from most published reports on resilience and brain reserve. First, we worked to strengthen our understanding of brain reserve by examining a broader set of dynamic biomarkers of brain reserve from multiple neuroimaging modalities. Traditional measures of brain reserve include premorbid brain volume, intracranial volume, or head circumference, which are fixed throughout the adult life (Stern et al., 2018). While these measures have been shown to be related to cognition, they are gross measures of overall brain reserve and do not encapsulate the likely modifiable nature of brain reserve. In this study, the static measure of intracranial volume that was used as a comparison region was only related to education-occupation and not to the other potential protective/risk factors that we identified. Whereas three of the four the dynamic brain reserve measures we used were associated with multiple general health factors. Importantly, our work builds upon recent studies that have started to expand our view of brain reserve with the incorporation of glucose metabolism, white matter integrity, and patterns of gray matter volume and cortical thickness (Querbes et al., 2009; Smith et al., 2010; Arenaza-Urquijo et al., 2013; Ewers et al., 2013; Morbelli et al., 2013; Malpetti et al., 2017; Pettigrew et al., 2017; Laubach et al., 2018). As shown in our results, the incorporation of carefully selected dynamic neuroimaging measures associated with cognitive aging may provide additional tools for the study of brain reserve; however, this will require future studies in independent samples.

Second, we studied a broad array of protective/risk factors that may impact brain health. Many studies on resilience use education, occupation, or lifestyle-social activity engagement as the sole proxy of cognitive resilience (Stern, 2009, 2012; Stern et al., 2018). While these contribute to the lower susceptibility to pathology, other factors may be additive in our understanding of cognitive resilience and brain health (Clare et al., 2017); notably: smoking, alcohol intake, and systemic vascular and metabolic health (Wolf et al., 1988; Ott et al., 1998; Thomas and Rockwood, 2001; Craft, 2009). The general health factors we examined were largely associated with lower brain reserve, as assessed by the four dynamic neuroimaging measures. By studying factors other than education, intellectual and physical activities as factors that influence cognitive resilience, we have the opportunity to better define which factors positively or negatively impact brain health and cognitive aging. This is of fundamental significance given the aging population throughout the world and an incomplete understanding of what factors lead to the complex, age-related cognitive disorders, like Alzheimer’s.

Third, our study was comprised of a late midlife sample (50–65 years) of participants without clinical signs of cognitive impairment. To date, many studies concerning resilience or brain reserve have focused on older adults. This is logical when working to assess resilience and brain reserve as pathologic differences may be more evident in that population; however, it is likely the brain changes that promote cognitive decline and age-related cognitive disorders begins earlier in life (see Figure 1). While we are currently unaware of exactly when these changes begin, pathology studies have shown very low prevalence of neurodegenerative and cerebrovascular pathologies before the age of 65 (Nelson et al., 2012). We would advocate for the study of protective/risk factors that influence resilience, brain reserve, and overall brain health throughout life to further our understanding.

Although it’s increasingly apparent that sex differences may impact brain health (Mielke et al., 2014; Chêne et al., 2015; Zagni et al., 2016), we still have an inadequate understanding of how sex impacts brain health throughout life and the propensity to develop age-related cognitive disorders. Although our study was not specifically designed to assess for sex differences, in our sample females scored higher on all cognitive domains except for visuospatial ability relative to age and education matched males. Moreover, despite having significantly lower total intracranial volume, females had no differences in AD region thickness, with higher superior parietal thickness and posterior cingulate FDG relative to males.

### Strengths and Limitations

The investigation of a narrow sample of the population (50–65 years) is a key strength of this work because this is a critical range where early brain and cognitive changes are observed without significant burden of cerebrovascular disease and neurodegenerative disorders. The large sample size (*n* = 537) with nearly identical number of females/males in late middle age that has been well characterized with demographic data and neuroimaging measures obtained at a single site strengthen our findings.

Several limitations include the homogeneity of our population-based sample relative to the United States and worldwide that may limit the applicability of findings. However, previous reports support the generalizability of our sample (Rocca et al., 2012; Sauver et al., 2012). The cross-sectional design of the study limits our ability to assess the relationship between protective/risk factors and brain reserve measures to the development of MCI, dementia, or cognitive change. While we found associations between brain reserve measures and cognition, these relationships will likely be best assessed in a longitudinal study where change in cognition and other clinical outcomes may be examined. Despite these limitations our study of a unique sample helps contribute to the understanding of cognitive aging and overall brain health in late midlife.

### Future Directions

Further work is needed to validate the findings of this study in an independent sample. Future studies will benefit from the development of composite risk scores and composite measures for brain health that can be used in tracking brain and cognitive aging throughout life. A longitudinal study would help to better assess cognitive decline (change in cognitive outcomes) and risk for development of MCI and dementia. It may be helpful to perform voxel-wise analyses of potential brain reserve measures. Lastly, we may work to stratify cognitively normal participants by CSF and/or PET amyloid and tau status to determine if this impacts the relationship between protective/risk factors and dynamic brain reserve measures. Interestingly, in a study of 52 cognitively normal participants, those with lower CSF amyloid and higher education had lower FDG PET uptake, while those with higher CSF amyloid and higher education had higher FDG PET uptake (Ewers et al., 2013). This suggests that protective factors may be differentially related to dynamic brain reserve measures depending on baseline amyloid burden.

**FIGURE 3:**
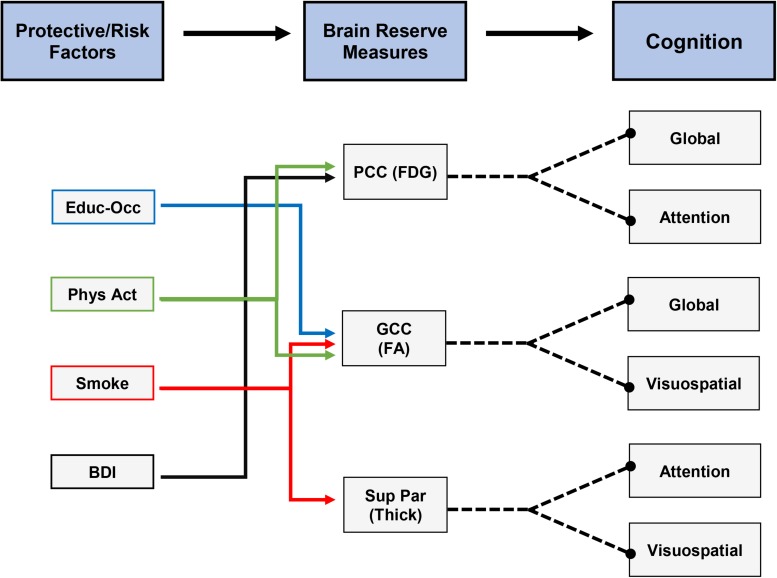
Hypothetical depiction of connections between protective/risk factors, brain reserve measures, and cognitive measures. All depicted relationships were significant in our analyses. Note: the cross-sectional design of our study limits the ability to directly connect each protective/risk factor to cognitive measures as mediated by individual brain reserve measures.

To better visualize potential connections between protective/risk factors, brain reserve measures, and cognition, we have compiled a flow diagram, indicating the significant relationships we found. Figure 3 is a hypothetical depiction of these relationships where both protective/risk factors and brain reserve measures were related to the same cognitive outcome. For example, being an ever-smoker was related to lower genu FA and lower superior parietal cortex thickness, which were related to worse global cognition, visuospatial ability, and attention. Thus, it may be possible for smoking to negatively affect cognition via impact on genu FA and superior parietal cortex thickness. As seen in this figure, future work would ideally focus on expanding our understanding of individual and combined factors that augment brain reserve measures and ultimately lead to discernable clinical outcomes (i.e., cognition, functional status). Further longitudinal analyses and data across the lifespan will allow us to understand the pathways proposed.

## Conclusion

In conclusion, we found that education-occupation was associated with cognition and the static brain reserve measure of total intracranial volume, but was not associated with any of the dynamic neuroimaging biomarkers. In contrast, many general health factors were associated with the dynamic neuroimaging proxies of brain reserve, while most were not associated with cognition in this late middle aged group. Brain reserve, as exemplified by the four dynamic neuroimaging features studied here, is itself at least partly under the influence of general health status in midlife, but remarkably is largely independent of education and occupation.

While an incomplete study of the factors that influence brain health and cognitive aging, this work contributes to the growing data that noticeable neuroimaging and cognitive relationships can be found in late midlife. We must continue to build a more comprehensive view of cognitive resilience and brain reserve to better understand the factors that make the brain vulnerable to age-related cognitive disorders.

## Data Availability Statement

The datasets generated for this study are available on request to the corresponding author.

## Ethics Statement

The studies involving human participants were reviewed and approved by the Mayo Clinic and Olmsted Medical Center Institutional Review Boards. The patients/participants provided their written informed consent to participate in this study.

## Author Contributions

BN conceptualized the study, was responsible for the analysis and owns primary authorship. JG-R conceptualized the study and critically revised the manuscript for intellectual content. MiM, CS, RR, VL, MS, MaM, RP, CJ, and DK critically revised the manuscript for intellectual content. SP and TL were responsible for the analysis and the critical revision of manuscript for intellectual content. PV conceptualized the study and owns primary authorship.

## Conflict of Interest

DK serves on a Data Safety Monitoring Board for the DIAN study, is an investigator in clinical trials sponsored by Biogen, Inc., Lilly Pharmaceuticals, and the University of Southern California. MiM served as a consultant to Eli Lilly and received unrestricted research grants from Biogen, Inc. and Lundbeck. VL consults for Bayer Schering Pharma, Piramal Life Sciences, and Merck Research and receives research support from GE Healthcare, Siemens Molecular Imaging, and AVID Radiopharmaceuticals. RP consults for Roche, Inc., Merck, Inc., Genentech, Inc., and Biogen, Inc., GE Healthcare, and Eisai, Inc., and receives royalties from Oxford University Press for the publication of Mild Cognitive Impairment. CJ consults for Lily and serves on an independent data monitoring board for Roche but he receives no personal compensation from any commercial entity. None of the relationships are relevant to the content in the manuscript. The remaining authors declare that the research was conducted in the absence of any commercial or financial relationships that could be construed as a potential conflict of interest.

## References

[B1] Arenaza-Urquijo E. M. Landeau B. La Joie R. Mevel K. Mezenge F. Perrotin A. (2013). Relationships between years of education and gray matter volume, metabolism and functional connectivity in healthy elders. *Neuroimage* 83 450–457. 10.1016/j.neuroimage.2013.06.053 23796547

[B2] Arenaza-Urquijo E. M. Vemuri P. (2018). Resistance vs resilience to Alzheimer disease: clarifying terminology for preclinical studies. *Neurology* 90 695–703. 10.1212/WNL.0000000000005303 29592885 PMC5894932

[B3] Beck A. T. Steer R. A. Brown G. K. (1996). *Manual for the Beck Depression Inventory-II.* San Antonio, TX: Psychological Corporation 490–498.

[B4] Bozzali M. Falini A. Franceschi M. Cercignani M. Zuffi M. Scotti G. (2002). White matter damage in Alzheimer’s disease assessed in vivo using diffusion tensor magnetic resonance imaging. *J. Neurol. Neurosurg. Psychiatry* 72 742–746. 12023417 10.1136/jnnp.72.6.742 PMC1737921

[B5] Byers A. L. Yaffe K. (2011). Depression and risk of developing dementia. *Nat. Rev. Neurol.* 7 323–331. 10.1038/nrneurol.2011.60 21537355 PMC3327554

[B6] Calle E. E. Thun M. J. Petrelli J. M. Rodriguez C. Heath C. W. Jr. (1999). Body-mass index and mortality in a prospective cohort of US adults. *N. England J. Med.* 341 1097–1105. 10511607 10.1056/NEJM199910073411501

[B7] Chêne G. Beiser A. Au R. Preis S. R. Wolf P. A. Dufouil C. (2015). Gender and incidence of dementia in the framingham heart study from mid-adult life. *Alzheimers Dement.* 11 310–320. 10.1016/j.jalz.2013.10.005 24418058 PMC4092061

[B8] Chua T. C. Wen W. Slavin M. J. Sachdev P. S. (2008). Diffusion tensor imaging in mild cognitive impairment and Alzheimer’s disease: a review. *Curr. Opin. Neurol.* 21 83–92. 10.1097/WCO.0b013e3282f4594b 18180656

[B9] Clare L. Wu Y.-T. Teale J. C. Macleod C. Matthews F. Brayne C. (2017). Potentially modifiable lifestyle factors, cognitive reserve, and cognitive function in later life: a cross-sectional study. *PLoS Med.* 14:e1002259. 10.1371/journal.pmed.1002259 28323829 PMC5360216

[B10] Coresh J. Selvin E. Stevens L. A. Manzi J. Kusek J. W. Eggers P. (2007). Prevalence of chronic kidney disease in the United States. *JAMA* 298 2038–2047. 17986697 10.1001/jama.298.17.2038

[B11] Craft S. (2009). The role of metabolic disorders in Alzheimer Disease and vascular dementia. *Arch. Neurol.* 66 300–305. 10.1001/archneurol.2009.27 19273747 PMC2717716

[B12] Crooks V. C. Lubben J. Petitti D. B. Little D. Chiu V. (2008). Social network, cognitive function, and dementia incidence among elderly women. *Am. J. Public Health* 98 1221–1227. 10.2105/AJPH.2007.115923 18511731 PMC2424087

[B13] Cunnane S. Nugent S. Roy M. Courchesne-Loyer A. Croteau E. Tremblay S. (2011). Brain fuel metabolism, aging, and Alzheimer’s disease. *Nutrition* 27 3–20. 21035308 10.1016/j.nut.2010.07.021 PMC3478067

[B14] Debette S. Seshadri S. Beiser A. Au R. Himali J. Palumbo C. (2011). Midlife vascular risk factor exposure accelerates structural brain aging and cognitive decline. *Neurology* 77 461–468. 10.1212/WNL.0b013e318227b227 21810696 PMC3146307

[B15] Ewers M. Insel P. S. Stern Y. Weiner M. W. Alzheimer’s Disease Neuroimaging Intitative. (2013). Cognitive reserve associated with FDG-PET in preclinical Alzheimer disease. *Neurology* 80 1194–1201. 10.1212/WNL.0b013e31828970c2 23486873 PMC3691784

[B16] Ewing J. A. (1984). Detecting alcoholism: the CAGE questionnaire. *JAMA* 252 1905–1907. 6471323 10.1001/jama.252.14.1905

[B17] Fortea J. Vilaplana E. Alcolea D. Carmona-Iragui M. Sánchez-Saudinos M. B. Sala I. (2014). Cerebrospinal fluid β-amyloid and phospho-tau biomarker interactions affecting brain structure in preclinical Alzheimer disease. *Ann. Neurol.* 76 223–230. 10.1002/ana.24186 24852682

[B18] Giorgio A. Santelli L. Tomassini V. Bosnell R. Smith S. De Stefano N. (2010). Age-related changes in grey and white matter structure throughout adulthood. *Neuroimage* 51 943–951. 10.1016/j.neuroimage.2010.03.004 20211265 PMC2896477

[B19] Jack CR. JR. Wiste H. J. Weigand S. D. Knopman D. S. Mielke M. M. Vemuri P. (2015). Different definitions of neurodegeneration produce similar amyloid/neurodegeneration biomarker group findings. *Brain* 138 3747–3759. 10.1093/brain/awv283 26428666 PMC4655341

[B20] Jagust W. (2013). Vulnerable neural systems and the borderland of brain aging and neurodegeneration. *Neuron* 77 219–234. 10.1016/j.neuron.2013.01.002 23352159 PMC3558930

[B21] Jefferson A. L. Himali J. J. Beiser A. S. Au R. Massaro J. M. Seshadri S. (2010). Cardiac index is associated with brain aging: the framingham heart study. *Circulation* 122 690–697. 10.1161/CIRCULATIONAHA.109.905091 20679552 PMC2929763

[B22] Johnson S. C. Christian B. T. Okonkwo O. C. Oh J. M. Harding S. Xu G. (2014). Amyloid burden and neural function in people at risk for Alzheimer’s disease. *Neurobiol. Aging* 35 576–584. 10.1016/j.neurobiolaging.2013.09.028 24269021 PMC4018215

[B23] Kochunov P. Williamson D. Lancaster J. Fox P. Cornell J. Blangero J. (2012). Fractional anisotropy of water diffusion in cerebral white matter across the lifespan. *Neurobiol. Aging* 33 9–20. 10.1016/j.neurobiolaging.2010.01.014 20122755 PMC2906767

[B24] Laubach M. Lammers F. Zacharias N. Feinkohl I. Pischon T. Borchers F. (2018). Size matters: grey matter brain reserve predicts executive functioning in the elderly. *Neuropsychologia* 119 172–181. 10.1016/j.neuropsychologia.2018.08.008 30102906

[B25] Le Bihan D. Mangin J. F. Poupon C. Clark C. A. Pappata S. Molko N. (2001). Diffusion tensor imaging: concepts and applications. *J. Magn. Reson. Imaging* 13 534–546. 11276097 10.1002/jmri.1076

[B26] Leech R. Sharp D. J. (2014). The role of the posterior cingulate cortex in cognition and disease. *Brain* 137 12–32. 23869106 10.1093/brain/awt162 PMC3891440

[B27] Malpetti M. Ballarini T. Presotto L. Garibotto V. Tettamanti M. Perani D. (2017). Gender differences in healthy aging and Alzheimer’s dementia: a 18F-FDG-PET study of brain and cognitive reserve. *Hum. Brain Mapp.* 38 4212–4227. 10.1002/hbm.23659 28561534 PMC6866811

[B28] Mielke M. M. Vemuri P. Rocca W. A. (2014). Clinical epidemiology of Alzheimer’s disease: assessing sex and gender differences. *Clin. Epidemiol.* 6 37–48. 24470773 10.2147/CLEP.S37929 PMC3891487

[B29] Morbelli S. Perneczky R. Drzezga A. Frisoni G. B. Caroli A. Van Berckel B. N. (2013). Metabolic networks underlying cognitive reserve in prodromal Alzheimer disease: a European Alzheimer disease consortium project. *J. Nucl. Med.* 54 894–902. 10.2967/jnumed.112.113928 23591639

[B30] Mosterd A. Hoes A. W. (2007). Clinical epidemiology of heart failure. *Heart* 93 1137–1146. 17699180 10.1136/hrt.2003.025270 PMC1955040

[B31] Nelson P. T. Alafuzoff I. Bigio E. H. Bouras C. Braak H. Cairns N. J. (2012). Correlation of Alzheimer disease neuropathologic changes with cognitive status: a review of the literature. *J. Neuropathol. Exp. Neurol.* 71 362–381. 10.1097/NEN.0b013e31825018f7 22487856 PMC3560290

[B32] Ott A. Slooter A. Hofman A. Van Harskamp F. Witteman J. Van Broeckhoven C. (1998). Smoking and risk of dementia and Alzheimer’s disease in a population-based cohort study: the rotterdam study. *Lancet* 351 1840–1843. 9652667 10.1016/s0140-6736(97)07541-7

[B33] Paulus W. J. Tschöpe C. Sanderson J. E. Rusconi C. Flachskampf F. A. Rademakers F. E. (2007). How to diagnose diastolic heart failure: a consensus statement on the diagnosis of heart failure with normal left ventricular ejection fraction by the heart failure and echocardiography associations of the european society of cardiology. *Eur. Heart J.* 28 2539–2550. 17428822 10.1093/eurheartj/ehm037

[B34] Pettigrew C. Soldan A. Zhu Y. Wang M.-C. Brown T. Miller M. (2017). Cognitive reserve and cortical thickness in preclinical Alzheimer’s disease. *Brain Imaging Behav.* 11 357–367. 27544202 10.1007/s11682-016-9581-y PMC5743433

[B35] Protas H. D. Chen K. Langbaum J. B. Fleisher A. S. Alexander G. E. Lee W. (2013). Posterior cingulate glucose metabolism, hippocampal glucose metabolism, and hippocampal volume in cognitively normal, late-middle-aged persons at 3 levels of genetic risk for Alzheimer disease. *JAMA Neurol.* 70 320–325. 23599929 10.1001/2013.jamaneurol.286 PMC3745014

[B36] Querbes O. Aubry F. Pariente J. Lotterie J.-A. Démonet J.-F. Duret V. (2009). Early diagnosis of Alzheimer’s disease using cortical thickness: impact of cognitive reserve. *Brain* 132 2036–2047. 10.1093/brain/awp105 19439419 PMC2714060

[B37] Ritchie K. Ritchie C. W. Yaffe K. Skoog I. Scarmeas N. (2015). Is late-onset Alzheimer’s disease really a disease of midlife? *Alzheimers Dement.* 1 122–130. 10.1016/j.trci.2015.06.004 PMC5975058 29854932

[B38] Roberts R. O. Geda Y. E. Knopman D. S. Cha R. H. Pankratz V. S. Boeve B. F. (2008). The mayo clinic study of aging: design and sampling, participation, baseline measures and sample characteristics. *Neuroepidemiology* 30 58–69. 10.1159/000115751 18259084 PMC2821441

[B39] Rocca W. A. Yawn B. P. Sauver J. L. S. Grossardt B. R. Melton L. J. (2012). History of the rochester epidemiology project: half a century of medical records linkage in a US population. *Mayo Clin. Proc.* 87 1202–1213. 10.1016/j.mayocp.2012.08.012 23199802 PMC3541925

[B40] Rohlfing C. L. Wiedmeyer H.-M. Little R. R. England J. D. Tennill A. Goldstein D. E. (2002). Defining the relationship between plasma glucose and HbA1c: analysis of glucose profiles and HbA1c in the diabetes control and complications trial. *Diabetes Care* 25 275–278. 11815495 10.2337/diacare.25.2.275

[B41] Sauver J. L. S. Grossardt B. R. Leibson C. L. Yawn B. P. Melton L. J. III Rocca W. A. (2012). Generalizability of epidemiological findings and public health decisions: an illustration from the rochester epidemiology project. *Mayo Clin. Proc.* 87 151–160. 10.1016/j.mayocp.2011.11.009 22305027 PMC3538404

[B42] Schwarz C. G. Gunter J. L. Wiste H. J. Przybelski S. A. Weigand S. D. Ward C. P. (2016). A large-scale comparison of cortical thickness and volume methods for measuring Alzheimer’s disease severity. *Neuroimage Clin.* 11 802–812. 10.1016/j.nicl.2016.05.017 28050342 PMC5187496

[B43] Sexton C. E. Walhovd K. B. Storsve A. B. Tamnes C. K. Westlye L. T. Johansen-Berg H. (2014). Accelerated changes in white matter microstructure during aging: a longitudinal diffusion tensor imaging study. *J. Neurosci.* 34 15425–15436. 10.1523/JNEUROSCI.0203-14.2014 25392509 PMC4228140

[B44] Smith C. D. Chebrolu H. Andersen A. H. Powell D. A. Lovell M. A. Xiong S. (2010). White matter diffusion alterations in normal women at risk of Alzheimer’s disease. *Neurobiol. Aging* 31 1122–1131. 10.1016/j.neurobiolaging.2008.08.006 18801597 PMC2873054

[B45] Stern Y. (2009). Cognitive reserve. *Neuropsychologia* 47 2015–2028. 10.1016/j.neuropsychologia.2009.03.004 19467352 PMC2739591

[B46] Stern Y. (2012). Cognitive reserve in ageing and Alzheimer’s disease. *Lancet Neurol.* 11 1006–1012. 10.1016/S1474-4422(12)70191-6 23079557 PMC3507991

[B47] Stern Y. Arenaza-Urquijo E. M. Bartrés-Faz D. Belleville S. Cantilon M. Chetelat G. (2018). Whitepaper: defining and investigating cognitive reserve, brain reserve, and brain maintenance. *Alzheimers Dement.* 10.1016/j.jalz.2018.07.219 [Epub ahead of print]. 30222945 PMC6417987

[B48] Stern Y. Chetelat G. Habeck C. Arenaza-Urquijo E. M. Vemuri P. Estanga A. (2019). Mechanisms underlying resilience in ageing. *Nat. Rev. Neurosci.* 20:246. 10.1038/s41583-019-0138-0 30814677

[B49] Thomas V. S. Rockwood K. J. (2001). Alcohol abuse, cognitive impairment, and mortality among older people. *J. Am. Geriatr. Soc.* 49 415–420. 11347785 10.1046/j.1532-5415.2001.49085.x

[B50] Traynor J. Mactier R. Geddes C. C. Fox J. G. (2006). How to measure renal function in clinical practice. *BMJ* 333 733–737. 17023465 10.1136/bmj.38975.390370.7C PMC1592388

[B51] Vemuri P. Lesnick T. G. Przybelski S. A. Graff-Radford J. Reid R. I. Lowe V. J. (2018). Development of a cerebrovascular magnetic resonance imaging biomarker for cognitive aging. *Ann. Neurol.* 84 705–716. 10.1002/ana.25346 30264411 PMC6282853

[B52] Vemuri P. Lesnick T. G. Przybelski S. A. Knopman D. S. Lowe V. J. Graff-Radford J. (2017). Age, vascular health, and Alzheimer disease biomarkers in an elderly sample. *Ann. Neurol.* 82 706–718. 10.1002/ana.25071 29023983 PMC5696029

[B53] Vemuri P. Lesnick T. G. Przybelski S. A. Knopman D. S. Preboske G. M. Kantarci K. (2015). Vascular and amyloid pathologies are independent predictors of cognitive decline in normal elderly. *Brain* 138 761–771. 10.1093/brain/awu393 25595145 PMC4339775

[B54] Vemuri P. Lesnick T. G. Przybelski S. A. Knopman D. S. Roberts R. O. Lowe V. J. (2012). Effect of lifestyle activities on Alzheimer disease biomarkers and cognition. *Ann. Neurol.* 72 730–738. 23280791 10.1002/ana.23665 PMC3539211

[B55] Vernooij M. W. Ikram M. A. Vrooman H. A. Wielopolski P. A. Krestin G. P. Hofman A. (2009). White matter microstructural integrity and cognitive function in a general elderly population. *JAMA Psychiatry* 66 545–553. 10.1001/archgenpsychiatry.2009.5 19414714

[B56] Whitmer R. A. Gustafson D. R. Barrett-Connor E. Haan M. N. Gunderson E. P. Yaffe K. (2008). Central obesity and increased risk of dementia more than three decades later. *Neurology* 71 1057–1064. 10.1212/01.wnl.0000306313.89165.ef 18367704

[B57] Whitmer R. A. Sidney S. Selby J. Johnston S. C. Yaffe K. (2005). Midlife cardiovascular risk factors and risk of dementia in late life. *Neurology* 64 277–281. 15668425 10.1212/01.WNL.0000149519.47454.F2

[B58] Wolf P. A. D’agostino R. B. Kannel W. B. Bonita R. Belanger A. J. (1988). Cigarette smoking as a risk factor for stroke: the framingham study. *JAMA* 259 1025–1029. 3339799

[B59] Yaffe K. Blackwell T. Whitmer R. A. Krueger K. Barrett Connor E. (2006). Glycosylated hemoglobin level and development of mild cognitive impairment or dementia in older women. *J. Nutr. Health Aging* 10 293–295. 16886099

[B60] Zagni E. Simoni L. Colombo D. (2016). Sex and gender differences in central nervous system-related disorders. *Neurosci. J.* 2016:2827090. 10.1155/2016/2827090 27314003 PMC4904110

[B61] Zhang Y. Schuff N. Jahng G.-H. Bayne W. Mori S. Schad L. (2007). Diffusion tensor imaging of cingulum fibers in mild cognitive impairment and Alzheimer disease. *Neurology* 68 13–19. 17200485 10.1212/01.wnl.0000250326.77323.01 PMC1941719

